# Impact of Exercise Prescription Education on Medical Student Confidence and Knowledge in Generating Exercise Recommendations

**DOI:** 10.7759/cureus.51396

**Published:** 2023-12-31

**Authors:** Gabrielle Pancio, Nathaniel Kern, Nethra Ankam, Xiao Chi Zhang

**Affiliations:** 1 Medicine, Thomas Jefferson University, Philadelphia, USA; 2 Rehabilitation Medicine, Thomas Jefferson University, Philadelphia, USA; 3 Emergency Medicine, Thomas Jefferson University, Philadelphia, USA

**Keywords:** physical activity guidelines, gamification, motivational interviewing, primary prevention, exercise prescription, exercise, physical activity, medical education

## Abstract

Despite physical activity being a key component of maintaining health and preventing disease progression, medical schools are not providing sufficient education on physical activity to medical students. As a result, medical students and new residents express a lack of confidence and knowledge when engaging in exercise prescription conversations with patients.

A group of 20 first-year medical students at Sidney Kimmel Medical College (SKMC) attended a selective course on exercise prescription and the American College of Sports Medicine (ACSM) physical activity guidelines. The course included gamification and case-based learning; students were introduced to fitness-related health issues and discussed how to adapt fitness guidelines for unique patient populations, including geriatric and cardiovascular patients. Cases were supplemented with gym equipment for students to further explore both aerobic and strength components of ACSM guidelines. Students’ confidence and knowledge of exercise prescription were assessed before and after the session via the Likert scale and case-vignette multiple-choice questions (MCQs), respectively. These surveys were also distributed to 18 SKMC first-year counterparts who did not participate in the course selection.

Based on survey scores, students' post-course self-reported confidence was significantly greater than before the session (p=0.034) and greater than that of students who did not participate in the course (p=0.005). Students’ knowledge increased and was significantly higher than that of course non-participants (p=0.018). This course highlighted that gamification and case-oriented education interventions can raise medical students' confidence in fitness in the hopes that they feel more comfortable providing exercise recommendations in the future.

## Introduction

Despite the fact that physical activity is a key component of maintaining health and preventing disease progression as it relates to cardiovascular disease, coronary artery disease, diabetes mellitus, and cancer [1-3], medical school education on physical activity guidelines is insufficient [4-5]. Medical students often express low confidence in engaging in conversations about fitness prescriptions with their patients [6-7] and family medicine interns report a lack of knowledge and skill in providing exercise recommendations [8]. Few incoming medical residents in family medicine, a crucial field for preventative care, report ever receiving a formal education in exercise, resulting in many providers feeling uncomfortable designing exercise goals for patients. This education gap is reflected in medical school curricula, as half of medical school curriculum directors agree they are not adequately preparing students for providing exercise recommendations to patients and the majority are not incorporating basic strength training guidelines in their didactic curriculum [9].

Despite the growing array of online education resources that address obesity counseling and other lifestyle competencies for medical students, there is limited publication on incorporating gamification and in-person fitness interaction to reinforce these topics. A review of the MedEd portal using keywords such as “exercise prescription,” “physical activity,” and “American College of Sports Medicine Activity Guidelines” revealed 10 similar resources that discussed motivational interviewing in generating fitness changes with patients or modules discussing nutrition and education guidelines. Current education modules cover a range of lifestyle competencies, but few are specific to exercise prescription and generating healthy lifestyle changes in patients.

Recognizing these curricular gaps, we developed and delivered a two-hour interactive course with the goal of educating students about physical activity guidelines to manage their own fitness goals and generate positive fitness changes while working with patients at our own institution more effectively. We designed this course to explore the effectiveness of different educational styles and the need for incorporating fitness education in preclinical curricula by using the combined instructional modalities of didactic presentations, small-group discussions, gamification, and physical instruction with gym equipment.

Educational objectives of the session

The session aims to achieve the following objectives: (i) understand the American College of Sports Medicine (ACSM) recommended activity guidelines for both aerobic and strength-building exercises for American adults and discuss exercise modalities to meet these guidelines; (ii) address modifiable risk factors for chronic disease and how they relate to exercise; (iii) engage with the concept of motivational interviewing, thereby understanding the transtheoretical model of behavior change; (iv) identify the four ways to target and modify exercise to fit various activity and body habit goals; (v) analyze individual barriers to fitness goals and outline actionable steps to combat these barriers.

## Materials and methods

We piloted our sweat science

The Basics of Exercise Prescription course was held in October of the 2022-2023 academic year at the Sidney Kimmel Medical College (SKMC) at Thomas Jefferson University during a two-hour Wellness Week session with 20 first-year medical students. Wellness Week is a dedicated week during the first semester of the first year’s curriculum where students engage in a series of lectures and various activities discussing mental, physical, and emotional wellness. During Wellness Week, students are able to sign up for a variety of offered courses. The inclusion criteria for course participants included currently matriculated first-year medical students at Sidney Kimmel College in good academic standing who were willing to participate in our wellness week course throughout its entire duration. There were no specific exclusion criteria for course participants.

The content of this two-hour course was developed and delivered by the authors of this study, two second-year medical students at SKMC, one of whom is a BOC-certified athletic trainer and NSCA-certified strength and conditioning specialist. The course was structured around the American College of Sports Medicine recommended activity guidelines for American adults, focusing on the guideline contents, how to adapt these guidelines to different disease states, and how to go about discussing topics of exercise prescription with patients. The style of the course was a combination of gamification, case-based style learning and discussion, and physical engagement with gym equipment. The course was instructed in the Jefferson Fitness Center, allowing for the dual nature of lecture style and physical education learning.

In order to analyze the effectiveness of the exercise prescription learning selective, students participated in a series of Qualtrics® surveys that we developed to evaluate both student confidence and knowledge in topics relating to physical activity and prescribing exercise recommendations to patients. The contents of the survey included a 5-point Likert scale and clinical vignette-style multiple-choice questions (MCQs) to assess confidence and knowledge. The survey utilized statements about students’ feelings of confidence in exercise prescription and in sharing this knowledge to encourage lifestyle change with their patients. Multiple-choice questions were designed to assess knowledge of exercise prescription principles, exercise physiology, and ACSM guidelines. There were a total of nine multiple-choice questions in the survey, with possible scores ranging from zero to nine based on the number of correct responses. Specifically, the content of the multiple-choice questions assessed students’ knowledge of the current ACSM guidelines. Current guidelines state that all healthy adults should participate in moderate-intensity aerobic physical activity for a minimum of 30 minutes on five days per week, or vigorous-intensity aerobic activity for a minimum of 20 minutes on three days per week and that every adult should perform activities that maintain or increase muscular strength and endurance for a minimum of two days per week [10]. We also assessed which gym equipment or exercises could be employed by patients to meet ACSM criteria, specifically comparing aerobic versus strength training tools. Lastly, we included questions exploring students’ understanding of expected physiologic changes during exercise, the implementation of motivational interviewing to enact change in patients’ fitness, and which disease states can be positively impacted by augmenting physical fitness.

Students participated in surveys both at the start of and directly prior to the Wellness Week course. Similarly, the pre-course survey was disseminated to 260 first-year counterparts who did not sign up for this Wellness Week course, yielding 18 responses. Results from both the Likert scale information and knowledge outcome score were input into Microsoft Excel for analysis. Mean data were compiled and pooled, utilizing a paired T-test for comparison amongst groups.

## Results

Twenty students from the first-year class at SKMC participated in our “Wellness Week” selective course on exercise prescription; 17 students (89%) completed the pre-course and post-course surveys. Students agreed that physical activity and exercise prescription are important factors at play in maintaining patient health before and after completion of the course (Table 1). In the post-session survey, students were more confident in their ability to discuss exercise goals and options with patients, including different exercise modality types and exercise prescription parameters. Students were more likely to want to incorporate physical exercise into their treatment plans for future patients following engagement with the session content (p=0.036).

**Table 1:** Ratings of intervention-related statements pre/post course surveys - rated on 5-point Likert scale. 1 = strongly disagree; 5 = strongly agree.

Item	Pre (n=17)	Post (n=17)	p-value
Physical activity and exercise prescription are important components of maintaining patient health.	4.83	4.89	
I am confident in my ability to provide exercise prescription recommendations to a patient.	3.27	4.05	
I am knowledgeable about different types of physical activity and exercise recommendations for different disease states.	3.33	4.05	
I am knowledgeable about the American College of Sports Medicine guidelines on physical activity.	2.16	4.33	
I am confident in my ability to discuss exercise options with patients.	3.39	4.26	
I plan to utilize exercise prescription as part of my treatment plan for future patients.	4.06	4.47	
Pooled Likert results	3.50	4.34	0.036

When comparing students who participated in this initiative to their first-year peers who did not (n=18), we found that following the course, participants were significantly more confident in their abilities to discuss exercise interventions and prescribe them to their patients compared to their matched counterparts (p=0.0049).

**Figure 1:**
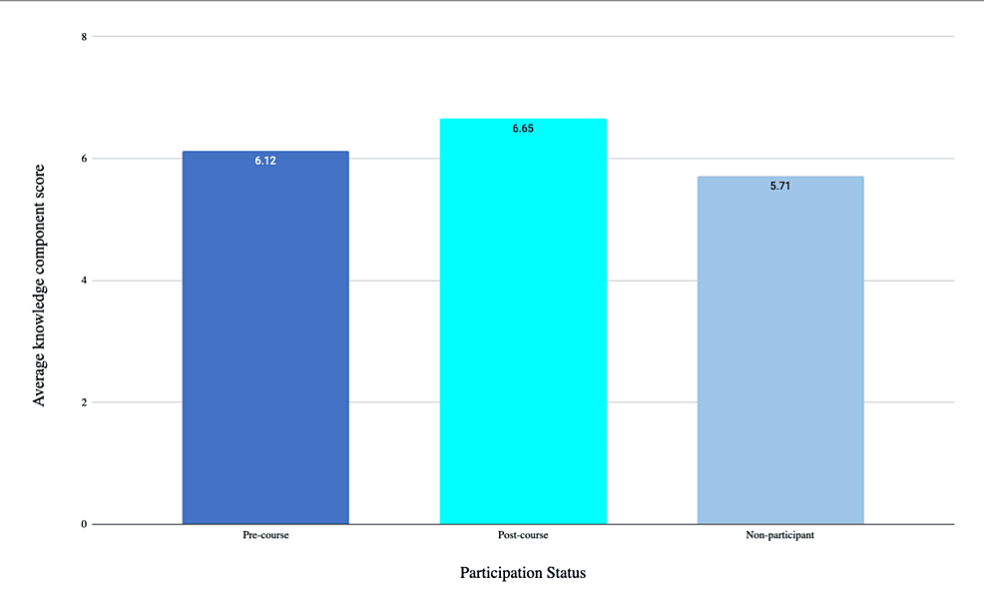
Average knowledge component scores pre- to post-course compared with non-participants. Possible scores ranged from zero to nine. An increase in the average knowledge component score pre- to post-course was non-significant (p=0.155). Comparisons between participants and non-participants showed a statistically significant difference in average knowledge component scores (p=0.018).

In analyzing the knowledge domain component of our survey, participants improved their scores on the multiple-choice style questions from an average of 67.9% to 74.8% (p=0.155) pre- to post-session (Figure 1). When comparing their knowledge base to the rest of their M1 counterparts, there was a significant difference in the knowledge gap. Course participants scored on average 6.55/9 following the session, compared to 5.71/9 for their match counterparts (p=0.018).

The results of the post-course survey were overwhelmingly positive, as all students agreed or strongly agreed the course would be beneficial to their future practice and the course received an average of 4.53/5 as a rating from the participants.

## Discussion

Our selective course intervention provided an evidence-based framework in strength and conditioning sciences for first-year medical students to utilize in their future patient care when incorporating physical activity for disease prevention or modification. Overall, our course was perceived as beneficial and was subjectively highly rated by participants. They enjoyed the gamification strategies employed throughout the session the interactive nature of the exercise coaching session and the interspersed discussion-based activities.

Students recognized the importance of physical activity and exercise prescriptions for maintaining health in their patient population before participating in the course. This belief was further solidified following course participation. We saw a significant increase in student confidence in fostering conversations about exercise and physical activity-based goals with patients and how to properly execute a plan of action to get their patients on the road to success. The didactic component of the session helped build student knowledge on proper ways to implement training protocols and the different types of exercise modalities that can be utilized to achieve both components of the ACSM guidelines. The course also explored disease-specific modifications that can be made to allow for the implementation of physical activity across a broad spectrum of patient health statuses and individualized patient goals.

When compared to their first-year peers who had not participated in this session, participants were significantly more confident in their abilities to navigate conversations surrounding exercise prescription with their patients and were objectively more knowledgeable about their ability to manipulate exercise parameters to benefit their future cohort of patients. Students who did not participate in our selection were not exposed to any formal training surrounding the concepts of exercise prescription in their pre-clinical years at SKMC. Highlighting the lack of exercise education provided in the preclinical curriculum at SKMC, course participants scored higher on the multiple-choice question (MCQ) component of the assessment than their peers who did not receive this training session, as expected. This is congruent with the work done by Recker et al. in 2021 [11], showing that medical students had a large gap in knowledge and confidence in this arena and that most of their knowledge of exercise prescription came from sources outside their formal medical training. The gap in knowledge between the two cohorts was indeed significant, highlighting that even one two-hour session on exercise prescription can facilitate learning on this important and clinically relevant topic.

This course offering took place during SKMC M1 Wellness Week, which is a full week off from didactic sessions following the second examination of students’ first year. One large strength of our session was the fact that students were relieved of educational and clinical responsibilities when they attended the selective course, allowing for more engagement with course content and less stress about their educational involvement. Our hands-on exercise coaching, as well as the engaging nature of a gamified session, were also noted to have a positive impact on the student experience. It could be of great benefit in the future to have other didactic sessions incorporate these interactive means to help reinforce lecture-based discussion. The use of gamification as a means of medical education is an evolving tool. As mirrored in other literature, our course highlighted that gamification has the potential to increase student motivation and engagement with learning objectives [12-15].

The greatest limitation to our intervention was time. In a two-hour session, it is impossible to sufficiently cover all the science and ideologies behind exercise prescription protocol; therefore, the content was compressed for students to form a framework to build on as they enter the clinical setting. Ideally, knowing how important physical activity is to the development and progression of copious disease processes, medical school curricula would begin implementing exercise physiology and physical activity prescription courses into their preclinical plans. Other limitations include the lack of a peer-reviewed and verified assessment tool when looking at student knowledge acquisition on these topics. Future work would include a standardized means of testing student knowledge in these domains so data can be compared across different types of interventions. Lastly, the potential for selection bias exists in the fact that students self-selected participation in this course, which could have resulted in individuals engaging with our content with a preexisting interest in exercise or an already-existing base of knowledge compared to their peers. This may have played a role in establishing the significant knowledge gap between our post-course participants and the rest of the first-year class. A self-selection bias also exists for course non-participants who completed our survey, given that only 18 of the 260 possible respondents answered the survey. A future investigation should be undertaken to assess baseline medical student knowledge on exercise prescription across multiple institutions to gain a more thorough understanding of where non-course participation knowledge lies.

Currently, we have evaluated only self-perceived confidence and knowledge across seven domains as well as objective knowledge across the MCQ assessment. Future research needs to be done to explore the retention of learning and the implementation of skills in patient practice. The ultimate educational goal of this course would be to increase clinical engagement with patients surrounding physical activity and the consistent incorporation of exercise intervention as a component of a comprehensive treatment plan.

We will continue to push for the integration of exercise physiology and physical activity prescription education into the medical school curriculum. Our study intervention shows that through engaging delivery of this content, we can help build physicians who show up to their clinical environments armed with the power of exercise as a critical tool to use to tackle an ever-complex medical landscape.

Our novel approach encouraged students to engage with current health guidelines and explore how to adapt recommendations for various patient populations. The overall purpose of the course was to help familiarize students with the exercise recommendations they will provide to patients as future providers and help improve their knowledge relating to their exercise.

## Conclusions

With dedicated, hands-on exercise prescription training, medical students will have more confidence discussing health and fitness with patients, helping patients identify areas for improvement and setting realistic, sustainable goals. Medical students will also be more skilled at engaging in exercise-related conversations with patients. Engaging in these conversations offers benefits to patients, as exercise and fitness-related intervention is an important component of primary prevention, and skilled providers can help to promote intrinsic motivation for health behavior change in their patients. Overall, increased utilization of the ACSM guidelines surrounding weight and fitness will improve health outcomes for patients across a variety of health domains.

## Appendices

Session workflow 

This module is designed to be delivered over a two-hour period.

Time: 0:00-0:20: Pictionary Icebreaker

The purpose of this icebreaker is to get students comfortable, engaged, and introduced to some of the ideas that will be discussed in the session in a fun and quick-paced way.

Students in groups of 4-5 were provided poster boards and markers to work through the following words and phrases, including words about fitness-related objects and activities and other, more challenging words surrounding physical health. Fitness objects and activities words:

Bike

Treadmill 

Broken bone

Running

Swimming

Squat 

Yoga

Bigger ideas surrounding physical health:

Fitness 

Mental health 

Strength 

Goals

Change 

Debrief

After wrapping up the activity, ask students if there were any words that were more difficult to draw. This will hopefully prompt students to discuss how some of the ideas relating to fitness were more difficult than the gym equipment and activity words. This is an opportunity to highlight how fitness and health goals can look different to every individual. This activity can help demonstrate to medical students that every patient will have unique goals, challenges, fitness levels, and ability to make changes.

Time 0:20-0:30: Introduce health as a concept and the ways in which the WHO’s top risk factors can be modified through exercise integration. Introduce the concept of the transtheoretical model of change and goal making. 

Time 0:30-0:35: Students work in groups of 4-5 to discuss times when goal attainment was successful and when it was not, what aspects of the model may have played a role in these endeavors.

Time 0:35-0:40: Students were tasked to form a goal utilizing the WOOP framework, with optional in-group sharing. 

0:40-0:42: Introduce ACSM guidelines for physical activity.

0:42-0:50: Utilize the “Crush the Crisis” framework to discuss osteoporosis as an introduction to muscle building exercise.

Crush the Crisis is a gamification medical education style developed in a workshop by Nethra Ankam and fellow Jefferson Medical Students. The objective of this learning game is for students to identify potential destabilizing factors in a patient's care and a practical plan for dealing with them. Demonstrate ability to provide first steps to deal with the crisis. 

0:50-1:10: Students were taken into the weight training facility to become familiarized with different modes of strength building exercise (machine weights, free weights, barbell weights, etc.). Students were then instructed on the barbell squat as a means to engage with physical activity themselves.

1:10-1:30: Didactic material delivered on strength building exercise selection, prescription, and assessment. 

Heavy emphasis placed on the manipulation of exercise selection, intensity, duration and frequency of exercise to fit a patient's individual performance, health and body structure goals.

130:-1:40: Utilize the “Crush the Crisis” framework to discuss the cardiovascular benefits of exercise.

1:40-1:45: Discuss amongst the cohort options for engaging in aerobic exercise and ways to modify those tasks to regress/progress patients. 

1:45- 1:55: Utilize the “Crush the Crisis” framework to discuss how to address getting patients started on an exercise program and how to maneuver possible roadblocks. 

1:55-2:00: Closing activity (Myth Buster): present common misconceptions or confusing topics in the physical activity space and have students decide their validity. 

END SESSION
